# Past, Present and Future Therapeutics for Cerebellar Ataxias

**DOI:** 10.2174/157015910790909476

**Published:** 2010-03

**Authors:** D Marmolino, M Manto

**Affiliations:** 1 Laboratoire de Neurologie Expèrimentale ULB-Erasme, Brussels, Belgium; 2 FNRS, Laboratoire de Neurologie Expèrimentale ULB-Erasme, Brussels, Belgium

**Keywords:** Cerebellum, ataxias, dominant, recessive, X-linked, therapy.

## Abstract

Cerebellar ataxias are a group of disabling neurological disorders. Patients exhibit a cerebellar syndrome and can also present with extra-cerebellar deficits, namely pigmentary retinopathy, extrapyramidal movement disorders, pyramidal signs, cortical symptoms (seizures, cognitive impairment/behavioural symptoms), and peripheral neuropathy. Recently, deficits in cognitive operations have been unraveled. Cerebellar ataxias are heterogeneous both at the phenotypic and genotypic point of view. Therapeutical trials performed during these last 4 decades have failed in most cases, in particular because drugs were not targeting a deleterious pathway, but were given to counteract putative defects in neurotransmission. The identification of the causative mutations of many hereditary ataxias, the development of relevant animal models and the recent identifications of the molecular mechanisms underlying ataxias are impacting on the development of new drugs. We provide an overview of the pharmacological treatments currently used in the clinical practice and we discuss the drugs under development.

## I. INTRODUCTION

With the advent of sensitive techniques such as magnetic resonance imaging (MRI), cerebellar ataxias (CAs) are being growingly recognized [11, 36, 57, 145, 165, 224]. There is a medical need to develop effective therapies in this group of disabling disorders for which no cure is currently available. For the first time, novel therapeutics aiming to target deleterious pathways are under development [100, 135, 214, 219]. The deciphering of the molecular mechanisms underlying CAs is a preliminary and unavoidable step to reach the goal of the cure [135]. Major advances in our current understanding of the pathogenesis of cerebellar ataxias have originated in particular from the development of relevant animal models which mimic the phenotype observed in human [90, 135, 166, 167, 173, 185]. Advances in brain neuroimaging have also improved drastically the early detection and follow-up of the course of cerebellar disorders [51, 77, 113]. This paper reviews the most common causes of CAs, the therapies used in the past and the treatments under assessment both at the experimental and clinical level. The main cerebellar disorders will be briefly presented for a matter of clarity.

## II. CEREBELLAR ATAXIAS: DESCRIPTION

CAs can affect the medial zone (vermal zone), the intermediate zone (paravermal zone) and/or the lateral zone of the cerebellum [133]. The main symptoms are listed in Table **1**. Cerebellar patients are typically clumsy during voluntary movements involving proximal or distal joints [64, 84, 209]. Cerebellum is also involved in several forms of motor learning [121, 125, 207]. CAs are a heterogeneous group of disabling disorders characterized by a lack of coordination and imbalance [114]. Patients exhibit various combinations of oculomotor deficits, dysarthria, dysmetria and kinetic tremor [138, 220]. Recently, the association of cerebellar lesions and neuropsychiatric symptoms has been underlined (Table **2**). It has been suggested that the cerebellum is involved in the pathogenesis of depression, schizophrenia and autism [58]. For instance, autism is associated with decreased densities of Purkinje neurons [124, 175, 228]. Some ataxic diseases present with marked cognitive dysfunction [181]. A typical example is SCA17, a highly heterogeneous disease with cognitive impairment in 80 to 100 % of cases [17, 65, 132, 239]. CAs can be divided in sporadic and inherited disorders [135]. Table **3** lists the principal disorders involving the cerebellum in young adults and for whom pharmacological treatments have been or are being evaluated [135].

### II.1. Cerebellar Circuitry: Composition, Neurotransmitters and Receptors

**Fig. (1):**
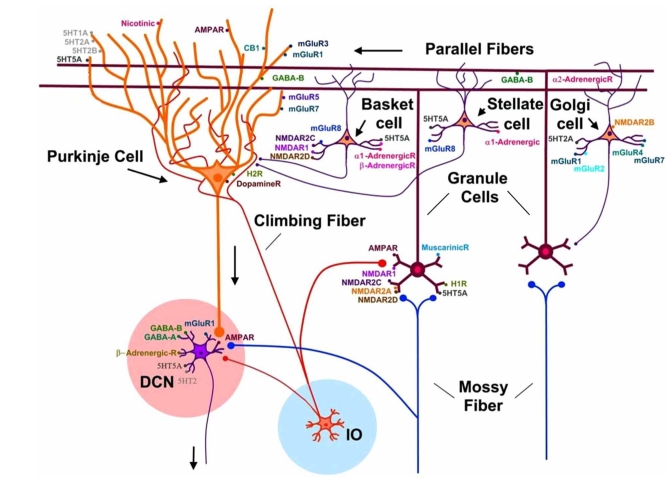
**Representation of the connectivity of cerebellar neurons and expression of receptors.** Two categories of inputs reach the cerebellar cortex: (1) the mossy fibers targeting granule cells and cerebellar nuclei (DCN), and (2) the climbing fibers originating from the inferior olivary complex (IO) and projecting to cerebellar nuclei and Purkinje neurons. Granule cells give rise to parallel fibers which make numerous synapses with dendritic spines of Purkinje cells. Inhibitory interneurons of the cerebellar cortex include basket cells, stellate cells and Golgi cells. Abbreviations: AMPA: aminohydroxymethylisoxazoleproprionate, NMDA: N-methyl-D-aspartate, mGluR. GABAA: GABA-A receptor, GABAB: GABA-B receptor, 5-HT: serotonin, CB1: cannabinoid 1 receptor.

Cerebellum is the region of the brain containing the highest number of neurons [132]. Cerebellar circuitry is highly complex. The cerebellum is composed of a mantle of grey zone (cortex) surrounding white matter, in which cerebellar nuclei are disposed [49]. Despite an apparent homogeneous structure, cerebellum is divided into several functional zones [208]. Three rostro-caudal longitudinal zones have been identified in the cerebellar cortex: a vermal zone projecting to the fastigial nucleus medially, an intermediate zone projecting to the interpositus nucleus and a lateral zone projecting to the dentate nucleus [73]. Two categories of inputs reach cerebellar cortex: mossy fibers issued from brainstem nuclei and climbing fibers originating from the inferior olivary complex [86]. Cerebellar cortex is composed of Purkinje cells, granule cells and inhibitory interneurons (Fig. **1**). Purkinje neurons exert an inhibitory effect upon cerebellar nuclei, which represent the sole output of cerebellar circuitry. There is increasing evidence of a functional asymmetry of the cerebellum, not only for motor but also for higher cognitive functions and sensory discrimination regarding processes like pain [18, 26, 85, 196, 232]. Another major feature of the cerebellar circuitry is the rostrocaudal and mediolateral subdivision of the cerebellar cortex into complex arrays of transverse zones and parasagittal stripes [3, 35, 82]. The most extensively studied marker for cerebellar compartmentation is the antigen zebrin II, an epitope on the respiratory isoenzyme aldolase C [156]. Zebrin II is expressed by a subset of Purkinje neurons forming parasagittal stripes which are highly reproducible between subjects and across species [156]. The molecular mechanisms which govern the distribution and organization of cerebellar zones are being unraveled and will probably lead to novel therapies [192]. 

Table **4** lists the neurotransmitters and neuromodulators of cerebellar circuitry. Distribution of the main receptors is illustrated in Fig. (**1**). Glutamate is a transmitter for both the mossy fiber and the climbing fiber system [191]. The postsynaptic element of the synapse mossy fiber-granule cell presents N-methyl-D-aspartate (NMDA) and aminohydroxymethylisoxazoleproprionate (AMPA) receptors [199]. Some mossy fibers contain choline acetyl transferase and use acetylcholine (Ach) as transmitter [110]. Climbing fibers are enriched in glutamate and can retrogradely transport aspartate to the inferior olive [94]. Glutamate is also the transmitter of parallel fibers which make numerous synapses with dendritic spines. Purkinje cell spines contain high densities of AMPA receptors [230]. Purkinje cells are enriched in glutamic acid decarboxylase (GAD), a GABA synthesizing enzyme [16]. Both ionotropic GABA receptors (GABA-A) and metabotropic GABA receptors (GABA-B) are highly expressed in the cerebellum. GABA-B receptors are particularly enriched in the cerebellar cortex [118, 137, 218]. At the cellular level, the GABA-B1 and GABA-B2 subunits are distributed in most neurons of the adult cerebellar cortex, but in particular at high levels in Purkinje neurons [105, 130].

Taurine is also enriched in Purkinje cells and is considered to play a role in osmoregulation [88]. Recent studies have demonstrated that retrograde messengers, the endocannabinoids, are released by Purkinje cells [179]. Type 1 cannabinoid receptors (CB1Rs) are expressed presynaptically at all synaptic inputs to the Purkinje neurons [206]. A high proportion of CB1Rs are also located at inhibitory interneurons throughout the molecular layer. Labelling studies are consistent with their presence in basket cell and stellate cell inhibitory interneurons [48]. 

GABA is the predominant transmitter of cerebellar interneurons (basket cells, stellate cells, Golgi cells, Lugaro cells). Glycine is also supposed to be involved in the inhibitory processes controlled by cerebellar interneurons [19]. The unipolar brush cells are an exception in the population of interneurons, since they are glutamatergic.

Aminergic fibers are found in the 3 layers of the cerebellar cortex (Table **5**). Serotoninergic fibers are distributed in all parts of the cerebellum, except lobule **X** [227]. Serotonin inhibits glutamate release from mossy fibers, tunes cellular responses to GABA and regulates the activity of glial GABA transporters [189]. Local application of serotonin modulates the firing rate of Purkinje cells both *in vivo* and *in vitro*. Lugaro cells are also responsive to serotonin [226]. The noradrenergic input modulates the responses of cerebellar neurons to GABA and glutamate. Interestingly, a genuine dopaminergic innervation is also found in the cerebellum [189]. Dopamine and dopamine transporters are found in all the layers of the cerebellar cortex, with the highest densities in the molecular layer [44, 162]. Dopamine immunoreactive axons are found in the cerebellar vermis of primates, primarily innervating the granule cell layer and the adjacent Purkinje neurons [89, 146]. Biochemical investigations have shown active dopamine uptake into cerebellar synaptosomes and release of endogeneous dopamine by the cerebellum [55, 70]. Radioligand binding studies have demonstrated that dopamine receptors 1-5, which belong to the D1-like and D2-like superfamilies, are expressed in the cerebellum [28, 45]. D1 and D5 receptors are localized in Purkinje neurons and the molecular layer, D2 receptors are found in all layers, D3 receptors are principally found in Purkinje neurons and molecular layer of lobules IX and X, and D4 receptors are concentrated in the molecular layer [70]. Levels of dopamine transporters (DAT) binding are a good indicator of the extracellular dopamine content. DAT bindings change in parallel to the concentrations of dopamine in the extra-cellular space [161]. DAT knockout mice (DAT -/-) are hyperactive [237]. Extra-cellular dopamine levels are increased in the striatum, causing a depression in mRNA levels of both D1 and D2 receptors and decreased stores of dopamine [71, 96]. Interestingly, several lines of evidence suggest that cerebellar dopaminergic system is a potential target of drugs of abuse [70]. Systemic administration of d-amphetamine and cocaine causes a dose-response increase in c-fos expression in the rat cerebellum, an effect which is reduced by pretreatment with SCH23390, a D1 receptor antagonist [59]. Moreover, a chronic treatment with cocaine sensitizes c-fos induction in the cerebellum *via *D1 and D2 receptors, and d-amphetamine increases the density of D2-like receptors [27, 39, 111]. Studies with cerebellar membrane preparations have shown that the [3H]GBR12935 specific binding is sensitive to mazindol, a specific dopamine transporter antagonist, and dopamine. Mazindol inhibits 15-20 % of the total specific binding with high affinity and the remaining 80-85 % with low affinity [44]. A complementary inhibitory pattern is observed with cis-flupentixol. Saturation experiments in presence of cis-flupentixol have revealed a specific binding site with a Kd value similar to the value in striatal preparations. Nevertheless, the affinity of dopamine for the [3H]GBR12935 specific binding is lower in the cerebellum, suggesting different three-dimensional conformations and ligand binding properties. 

The hypothalamus projects to the cerebellum using histamine as transmitter [50]. Histaminergic fibers are found in all cortical layers of the cerebellum. H1 receptors are preferentially expressed on Purkinje cell dendrites [213].

Activation of NMDA receptors in the cerebellum stimulates the production of nitric oxide (NO) [60]. Granule cells are a main source of NO [131]. Granule cells, parallel fibers and basket cells are equipped with the neuronal isoform of NO synthase (nNOS). Release of NO from parallel fibers is involved in long-term depression (LTD) [157]. Production of cyclic guanosine monophosphate (cGMP) results indirectly from NO production [76]. cGMP immunoreactivity is found mainly in Bergmann glia and astrocytes [198]. 

### II.2. Inherited CAs

The inherited ataxias are related to a genetic deficit (Table **6**). They can be divided into four groups: autosomal dominant ataxias, autosomal recessive ataxias, mitochondrial ataxias and X-linked ataxias [14, 112, 115, 135, 159]. 

#### II.2.1. Autosomal Dominant Ataxias (ADCAs)

Onset varies from infancy to the elderly. In most cases, symptoms start between the ages of 20 and 60 [135]. 

##### Spinocerebellar Ataxias (SCAs)

SCAs are a set of genetic and clinically heterogeneous diseases which share the feature of progressive ataxia [135, 184]. They have an incidence of 1 to 4 affected every 100.000 [215]. SCAs are classified genetically according to a specific mutation or mapped locus, and also according to clinical findings [207]. Dentatorubropallidoluysian atrophy (DRPLA) and ataxia associated with fibroblast growth factor 14 (FGF14) mutation are also considered in this group [136].

The majority of known mutations involve a sequence of CAG trinucleotide repeats within the coding tract in the respective gene. This is the case for SCA1, SCA2, SCA3, SCA6, SCA7, SCA17, and DRPLA, while a CTG repeats is present in SCA8 [207]. Trinucleotide repeats are associated with an abnormal polyglutamine accumulation and formation of nuclear aggregates [155]. A pentanucleotide repeat expansion ATTCT is associated with SCA10 [142]. SCA5, SCA13, SCA14, and 16q22-linked autosomal dominant cerebellar ataxia (ADCA) are characterized by point mutations [108, 147, 158, 229]. Patients exhibit various combinations of cerebellar and extra-cerebellar deficits (Table **7**).

#### II.2.2. Episodic Ataxias (EAs)

EAs are a group of diseases underlying a monogenic mutation (EA 1-7; Table **8**) [102]. The onset of symptoms is generally in the childhood [100]. Anxiety and fatigue increase the susceptibility to an attack [30]. EA-2 is the most common episodic ataxia [34]. Patients may exhibit myokymia, nystagmus, vertigo, and ataxia [101, 169]. EA-1 is characterized by short attacks of incoordination and dysarthria (slurred speech). 

#### II.2.3. Autosomal Recessive Ataxias (ARCAs)

ARCAs are disorders characterized by variable combinations of central and peripheral nervous system involvement [57]. Dysfunctions of other organs are found in some ARCAs [220]. The most common ARCA is Friedreich’s ataxia (FRDA). Other recessive ataxias include ataxia-telangiectasia, ataxia with ocular motor apraxia (AOA), ataxia with vitamin E deficiency (AVED), ataxia with CoQ10 deficiency, abetalipoproteinemia, early-onset cerebellar ataxia with retained tendon reflexes, infantile onset spinocerebellar ataxia, Marinesco-Sjogren syndrome and spastic ataxia of Charlevoix-Saguenay [30, 135]. There are numerous additional types of recessive ataxias worldwide, identified in a few families only. The most common ARCAs are briefly described below.

**Friedreich’s ataxia (FRDA)** is due to a pathological GAA triplet expansion within the first intron of the FXN gene, codifying for the mitochondrial protein frataxin [32, 140]. It is a severe neurodegenerative disorder, with an incidence of 1/40.000 [43]. The clinical picture is characterized by gait and limb ataxia, dysarthria, areflexia, proprioceptive loss and Babinski sign [62]. Patients can also develop cardiomyopathy and diabetes mellitus [62]. There is a consensus that FRDA is a disorder of iron homeostasis at the mitochondrial level [66, 115].

Cerebellar ataxia with muscle **Coenzyme Q10 deficiency** is a heterogeneous disorder. The cerebellar form presents with ataxia and cerebellar atrophy [148, 150, 122]. Coenzyme Q10 (CoQ10; ubiquinone), is an electron carrier critical for electron transfer within the mitochondrial respiratory chain (see section IV.2).

Ataxia with **Vitamin E deficiency (AVED), **presents as a FRDA-like syndrome [6, 20]. The 744deletionA is the most common mutation. Abetalipoproteinaemia (ABL) is characterized by a deficit in the low density apolipoprotein-B (VLDLs). Symptoms are similar to AVED.

**Ataxia-telangiectasia** is the most frequent ARCA in children [67, 68]. First symptoms usually begin between 2-8 years. A mutation within the ATM gene results in a deficit in the DNA repair [193]. 

Ataxia with **oculomotor apraxia (AOA)** type 1 is due to a mutation of the aprataxin gene and is associated with hypoalbuminemia [41, 42]. AOA type 2 is due to a mutation in the senataxin gene [10]. AoA2 is associated with increased alpha-fetoprotein levels in blood [123].

**Late-onset Tay-Sachs disease (LOTSD)** is characterized by a deficiency in beta-hexosaminidase due to a mutation in the HEXA gene [149]. Patients presents a FRDA-like phenotype.

**Cerebrotendinous xanthomatosis (CTX)** patients carry a mutation within the mitochondrial enzyme sterol 27-hydroxylase (CYP27). Serum analysis shows increased levels of cholestanol and bile alcohols [202]. Patients exhibit combinations of dementia, psychiatric disturbances, pyramidal deficits, extra-pyramidal deficits (dystonia), cerebellar signs, seizures, and peripheral neuropathy [192]. Neuropsychiatric symptoms such as hallucinations, agitation, depression, and suicide attempts may be prominent. Infantile-onset diarrhea, cataract and tendon xanthomas are suggestive [223].

**Refsum’s disease** is caused by mutation of the gene for the peroxisomal enzyme phytanoyl-CoA hydroxylase, PHYH [99]. The age of onset varies from early childhood to 50 years of age. Most patients have symptoms before age of 20 years. The main clinical features are retinitis pigmentosa, chronic polyneuropathy, and cerebellar ataxia [109]. Anosmia, sensorineural deafness, cardiac arrhythmias, renal failure, bony and skin abnormalities have been reported [126]. 

**SCA with axonal neuropathy (SCAN1)** is due to a mutation within the TDP1 gene, encoding for the tyrosyl-DNA phosphodiesterase 1 [56]. SCAN1 is characterized by peripheral sensori-motor axonal neuropathy, distal muscular atrophy, and pes cavus. Patients exhibit steppage during gait. The phenotype can mimick Charcot-Marie-Tooth disease. Patients have a history of seizures.

**The recessive spinocerebellar ataxia type 1 (SCAR8; ARCA1) **maps to locus 6q25. The disease is caused by mutations in the SYNE1 gene [54]. SCAR8 was initially identified in French Canadian families originating mainly from the Beauce and Bas-St-Laurent regions of the province of Quebec (Canada). Patients present a late-onset cerebellar ataxia with slow progression [74]. They exhibit dysarthria, dysmetria, occasional brisk lower-extremity tendon reflexes, and minor oculomotor abnormalities. Brain MRI shows a diffuse pure cerebellar atrophy. 

**Autosomal recessive spastic ataxia of Charlevoix-Saguenay (ARSACS)** is due to a deficit in the chaperone protein sacsin (SACS) [222]. The age of onset ranges from 1 to 14 years [29]. ARSACS is characterized by early-onset spastic ataxia, axonal and demyelinating neuropathy, and hypermyelination of retinal nerve fibers [205]. Pes cavus is common. Gait unsteadiness is the first symptom in most cases. A minority of patients develop seizures. Intelligence is usually normal. 

In the **infantile-onset spinocerebellar ataxia (IOSCA)** the gene C10orf2 encoding for the protein twinkle, a mitochondrial helicase involved in DNA replication, is mutated [154]. The disease has been described in Finland and is characterized by a very early onset ataxia (between 1 and 2 years), athetosis and tendon hyporeflexia. Ophthalmoplegia, hearing loss, and sensory neuropathy appear later in the disease course. Refractory status epilepticus, migraine-like headaches and severe psychiatric symptoms are also suggestive [128]. 

**Marinesco-Sjögren syndrome (MSS)** is due to a mutation in the chaperone protein HSPA5 transcribed by the gene SIL1 [5]. Symptoms of MSS start in the infancy [221]. Manifestations include cerebellar ataxia, congenital cataracts, retarded somatic and mental development, muscle weakness, hypotonia and tendon areflexia. 

**Wilson disease**, a treatable disorder resulting from copper accumulation and subsequent hepatic dysfunction, has variable presentations. Cerebellar symptoms may be present and tremor appears in up to 50% of patients. The Kayser-Fleischer ring (a deep copper-colored ring at the periphery of the cornea, the color ranging from greenish gold to brown) is observed in up to 90% of individuals and is almost invariably present in patients with neurologic manifestations. Low ceruloplasmin levels are found in blood. Levels of total copper are decreased. Hemolytic anemia (Coombs-negative) occurs in 10-15 % of cases.

#### II.2.4. Mitochondrial Disorders

This group gathers diseases due to mutations in mitochondrial genes. Most genes are involved in the energy production, essentially in oxidative phosphorylation [238]. Some mitochondrial disorders have ataxia as a main symptom, such as Kearns-Sayre Syndrome, May-White Syndrome, MNGIE (ophthalmoparesia, peripheral neuropathy, and gastrointestinal symptoms), Leigh syndrome, NARP (neuropathy, ataxia, and retinitis pigmentosa), MELAS (mitochondrial encephalomyopathy, lactic acidosis with stroke-like episodes), and MEERF (myoclonus epilepsy with ragged red fibers) [63]. Mutations within the mitochondrial DNA polymerase (POLG) catalytic subunit γ are associated with a progressive external ophthalmoplegia and hepatocerebral disturbances [33]. 

#### II.2.5. X-linked Inherited Ataxias

The commonest X-linked ataxias are listed in Table **9**. Fragile-X tremor ataxia syndrome (FXTAS) is rare disease in which a CGG mutation is carried on the X chromosome [97, 98]. The disorder usually starts after 50 years. Typical symptoms are gait ataxia, kinetic tremor, parkinsonism, polyneuropathy, and cognitive dysfunctions [2, 21]. 

## III. SPORADIC ATAXIAS 

This class of ataxias can be divided in (1) degenerative and (2) acquired ataxias. Degenerative ataxias include multiple system atrophy (MSA) and idiopathic late-onset cerebellar ataxia (ILOCA) [69]. MSA is a progressive adult-onset disease. A cerebellar form (c-MSA) and a parkinsonian form (p-MSA) are observed. Patients exhibit dysautonomia. 

Acquired ataxias are listed in Table **10**. The drugs which can trigger a cerebellar syndrome are given in Table **11**. The most common cerebellotoxic agent is alcohol. Chronic consumption leads to cerebellar atrophy which is most pronounced in the anterior vermis [95]. The principal neuropathological findings are a loss of Purkinje neurons and a drop in the dendritic network in the molecular layer [163]. By contrast, granule cells are relatively resistant to chronic ethanol exposure [203]. Excitotoxicity contributes to ethanol/withdrawal-induced lesions in the cerebellum [129]. Excitatory signaling outweighs inhibitory transmission. There is an excessive glutamate binding to NMDA receptors, followed by calcium influx into neuronal cytoplasm. Although acute doses of ethanol inhibit the NMDA-mediated toxicity of granule cells in culture, chronic administration enhances the NMDA receptor activity [91, 92]. Local administration of ethanol in cerebellar nuclei *in vivo* decreases extracellular levels of GABA and increases the NMDA-induced production of NO [134]. Chronic exposure of Purkinje neurons to ethanol increases the AMPA-elicited calcium influx [153]. Acute doses of ethanol act also directly on gabaergic pathways. In particular, acute doses potentiate the function of GABA-A receptors, explaining partly the sedative and anti-anxiety effects [119]. Chronic ethanol exposure depresses the expression of GABA-A receptors alpha1-subunit and increases alpha6-subunit expression, mediating the development of tolerance to the motor-impairing effects of ethanol [119]. Another mechanism of cerebellar toxicity of ethanol is linked to the effects on thiamine, an essential cofactor of alpha-ketoglutarate dehydrogenase, pyruvate dehydrogenase and transketolase, 3 key-enzymes of energy metabolism and lipid synthesis in the brain [95]. The effects of thiamine deficiency on diencephalic lesions in Wernicke encephalopathy are well demonstrated [112]. Cerebellum is the site of the brain with the highest turn-over rate of thiamine, which could explain the particular vulnerability of the cerebellum to thiamine deficit. Several studies have also underlined the relationship between ethanol and oxidative stress. Ethanol-induced oxidative stress contributes to the observed apoptotic neuron loss, with accumulation of 4-hydroxynonenal (HNE; see section IV), a toxic product of lipid peroxidation which accumulates in ethanol-exposed brain mitochondria and triggers a release of apoptosis-inducing factor from mitochondria in a dose-dependent manner [168]. 

Immune-mediated ataxias include: multiple sclerosis, cerebellar ataxia with anti-glutamic acid decarboxylase (GAD) antibodies, gluten ataxia, Miller-Fisher syndrome, systemic lupus erythematosus, Sjögren syndrome, Cogan syndrome, thyroiditis [7, 15, 75, 180, 190]. In children, tumors causing ataxic syndromes include medulloblastomas, astrocytomas, and ependymomas [143]. In adults metastatic tumors and hemangioblastomas are the most common cerebellar neoplasms. Paraneoplastic ataxias often present as a subacute cerebellar syndrome, sometimes mimicking a cerebellitis [104]. Patients may present with neurologic symptoms before identification of the underlying tumor. Paraneoplastic cerebellar degeneration is often mediated by antibodies usually generated against various tumor antigens (in particular anti-Yo/anti-Hu/anti-Ri/ Anti-mGluR1/Anti-Zic4/anti-CV2 antibodies). The most commonly associated cancers involve the ovary, uterus, breast, lungs, or ataxia may be associated with Hodgkin lymphoma.

## IV. TREATMENTS OF CAS

### IV.1. Current Symptomatic Therapy

Several treatments, mainly targeting neurotransmitters, have been assessed these last decades. It should be pointed out that 4 barriers have hampered meaningful clinical trials [219]: the rarity of each cerebellar disorder considered alone, the heteregeneous presentation of CAs, the fact that a substantial degree of neuronal loss has already occurred when symptoms appear, and the absence of biomarkers. However, some CAs do respond to specific therapies and should not be overlooked, such as AVED which responds to vitamin E supplements [160]. We briefly summarize below the current general management of cerebellar disorders. 

#### General Recommandations

Speech rehabilitation and regular physiotherapy/occupational therapy are recommended in CAs. Most patients have some improvements with the use of orthosis, sticks, or strollers. Unfortunately, many patients will become wheelchair bound during the course of their illness. Gastrostomy is usually recommended when swallowing difficulties worsen.

##### Pharmacotherapy

The principal drugs which have been suggested for treatment of nystagmus are gabapentin (a GABA analogue), clonazepam, 3, 4-diaminopyridine, baclofen and memantine (an uncompetitive NMDA antagonist). Action tremor may respond to primidone, beta-adrenergic blocking agents, such as propanolol, and to benzodiazepines [135]. Appropriate medications may be given for associated symptoms such as spasticity, parkinsonism, dystonia, bladder dysfunction, and orthostatic hypotension. In particular, parkinsonian symptoms may improve with levodopa or dopamine agonists. Spasticity is treated with baclofen/tinazidine. Alternatives include benzodiazepines and tizanidine. Dystonia may respond to diphenhydramide, benzotropine mesylate, or biperiden. Seizures are treated with conventional antiepileptic medications. Myoclonus may be attenuated with benzodiazepines, high doses of piracetam or sodium valproate. For urinary urgencies, the use of spasmolytics or adrenergic a-receptor blockers is effective. Hypotension may respond to increasing salt in the diet or may subside with the use of fludrocortisone or midodrine.

##### Surgical Therapy

High-frequency electrical stimulation (DBS) of the ventral intermediate nucleus (Vim), or surgical lesions (thalamotomy), can reduce cerebellar tremor. However, there is usually no effect on dysmetria.

##### Treatment of Paraneoplastic Cerebellar Ataxia

The treatment of the primary cancer should be performed as soon as possible in paraneoplastic cerebellar ataxia, hence the importance of early detection. Various combinations of surgery, chemotherapy and radiotherapy are considered according to the neoplasm. Intravenous immunoglobulins and plasmaphereses have been used in some cases with variable results. The role of intravenous steroids is not established.

##### Autosomal Dominant Cerebellar Ataxias (ADCAs)

In case of neurodegeneration as observed in SCAs, none of the symptomatic therapies mentioned above has stopped the progression of the disease, although symptomatic improvements have been reported (Table **11**). Slight benefits have been observed using 5-hydroxytryptophan, buspirone or tandospirone, sulfamethoxazole/trimethoprim or lamotrigine. Acetazolamide may decrease the ataxic symptoms in SCA6. Muscle cramps may be disabling, especially in SCA3, and may respond to magnesium, quinine, or mexiletine [52, 160, 233]. 

##### Episodic Ataxias

The main treatment for episodic ataxia (EA1) is acetazolamide [81]. 4-aminopyridine, phenytoin and cabamazepine are alternatives. Phenytoin and carbamazepine may exacerbate symptoms in EA2. 

##### Recessive Ataxias

In FRDA patients, a monitoring for cardiomyopathy and diabetes yearly is important [188]. Idebenone (2, 3-dimethoxy-5-methyl-6-(10-hydroxydecyl)-1, 4-benzoquinone) has been shown to reduce cardiac hypertrophy in most patients, but does not stop progression of ataxia [46, 139, 164, 177, 211]. Iron chelators, such as desferoxiamine and deferiprone could have beneficial effects [72, 127, 195]. Recombinant human erythropoietin (EPO) and peroxisome proliferator activated receptor gamma agonists (PPARgamma) are under investigation (see section IV.2) [1, 25, 79, 141, 201]. Beta-blockers at high doses could provide benefits in patients with heart involvement. 

In AVED, oral supplementation of vitamin E is the treatment of choice to improve the clinical status [186]. ABL is also treated with vitamin E. CoQ10 supplementation improves ataxia in case of deficiency of CoQ10. Treatment of CTX includes oral administration of chenodeoxycholic acid and statins such as pravastatin to inhibit hydroxylmethylglutaryl (HMG)-CoA reductase. Refsum’s disease is treated with dietary restriction of phytanic acid. 

##### Sporadic Ataxias

Specific intoxications and endocrine ataxias should be treated with specific therapies. Vitamin B1 supplementation is recommended in cerebellar degeneration due to alcoholic intoxication [95]. Abstinence is mandatory to avoid the progression of the degeneration.

In gluten ataxia, the benefits of a gluten-free diet in the treatment of patients with celiac disease are well established [75]. The most reliable marker of adherence to a gluten-free diet is serological evidence of elimination of circulating anti-gliadin antibodies [75]. Patients with gluten ataxia in absence of enteropathy may improve with intravenous immunoglobulins. Treatment with immunosuppressants should be considered if strict gluten-free diet has not resulted in improvement of ataxia after a year or if the ataxia is rapidly progressive [75].

Medications used to slow the progression of multiple sclerosis include immune modulators such as interferons, monoclonal antibodies, glatiramer acetate, mitoxantrone, methotrexate, azathioprine, cyclophosphamide, and natalizumab. Steroids may be used to decrease the severity of attacks. It is still unclear whether i.v. (intravenous) immunoglobulin and plasmapheresis improve patients’ outcomes in Miller-Fisher syndrome [151]. Steroids are also useful for the management of raised intra-cranial pressure associated with cerebellar disorders and for post-infectious cerebellitis [236]. 

##### Mitochondrial Disorders

**Fig. (2):**
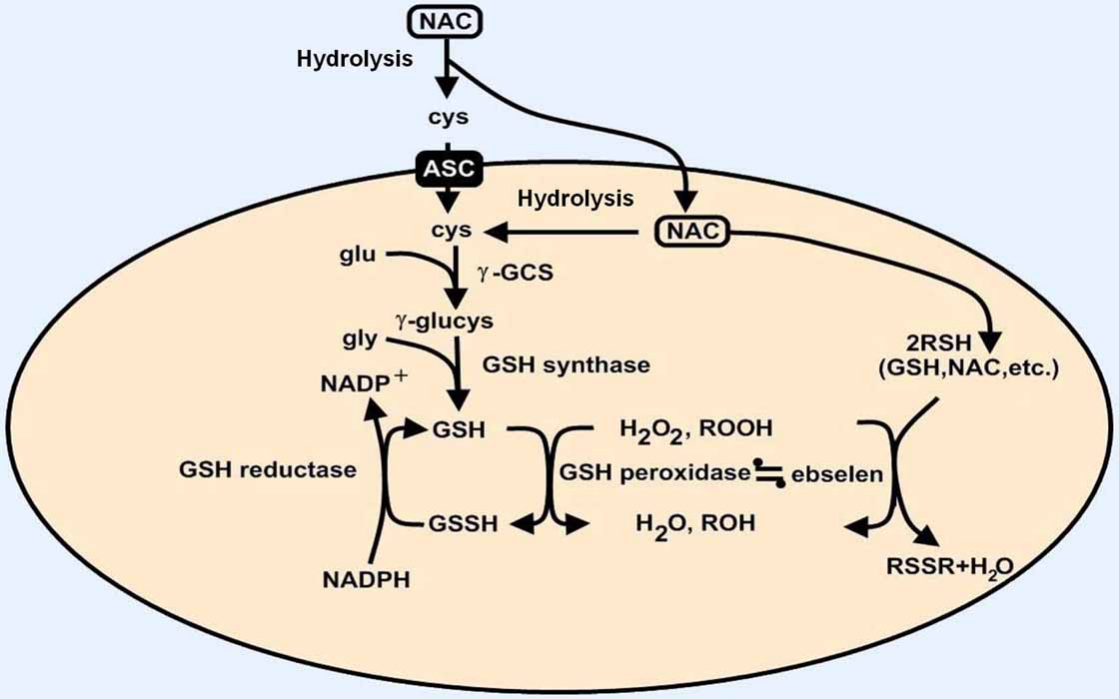
**Mechanism of action of N-acetylcysteine (NAC).** ASC, alanine-serine-cysteine (ASC) transport system; c-GCS, c-glutamylcysteine synthetase; cys, cysteine; glu, glutamine; gly, glycine; GSH, glutathione. Adapted from Arakawa and Ito (2007), [9].

High doses of CoQ10 and N-acetylcysteine (NAC), which was considered as one of the most promising drug, are used in maternally inherited mitochondrial diseases, but their efficacy is not established. Oxidative stress decreases cellular bioenergetic capacity, which will generate reactive oxygen species (ROS) [9]. Antioxidants such as NAC are involved in glutathione regeneration (Fig. **2**). Cysteine is transported by the alanine-serine-cysteine (ASC) system, but NAC does not require any active transport to deliver cysteine to the cell [12]. NAC is rapidly hydrolyzed and under the consecutive effects of gamma-glutamylcysteine synthetase and GSH synthetase, GSH is generated. This synthesis is highly limited by the availability of the substrates. Gamma-glutamylcysteine synthetase is inhibited by feedback from GSH (Ki around 1, 5 mM). Therefore, this enzyme is not operating at its maximal rate under physiological conditions. Intracellular GSH is maintained in the thiol form by the gluthatione reductase, which requires NADPH. GSH can react with non enzymatically with ROS, and GSH peroxidase catalyzes the destruction of hydrogen peroxide and hydroperoxides. From a pharmacokinetic point of view, infused NAC rapidly generates disulfides in plasma, which prolongs the existence of the drug from a few minutes to up to 6 hours. Nevertheless, free thiol is undetectable following oral ingestion of a dose of 200 mg of NAC. The bioavailability of the oral formulation is less than 5 %. This factor contributes to the failure of NAC given orally to prevent the raise in ROS in neurodegenerative disorders like CAs.

**Fig. (3):**
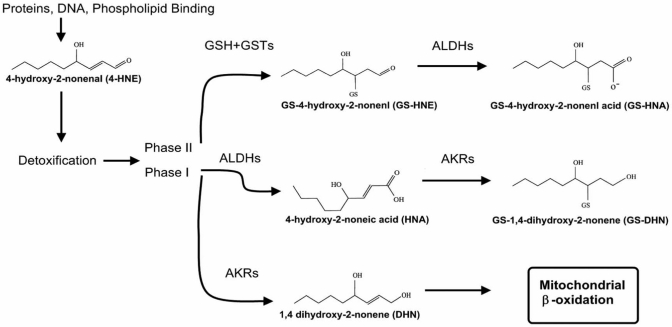
**Potential routes of mitochondrial HNE metabolism.** HNE is able to alkylate diverse classes of biological molecules. Balancing this toxicity is the metabolism of HNE by multiple phase I and phase II pathways. GS-HNE and GS-HNE acid can dehydrate to form a cyclic hemiacetal and lactone, respectively. Adapted from Arakawa and Ito 2007, [9].

Membrane lipid peroxidation results from accumulation of ROS. One of the products of lipid peroxidation is HNE (Fig. **3**), which is associated with inhibition of the activity of several types of cellular function and signaling, and exhibits cytotoxicity through alkylation [22, 106]. High levels of HNE have been found in the brain of patients with Alzheimer’s disease (AD). Increased HNE levels have also been found in the cerebellum of patients with spinocerebellar degeneration [235]. HNE-induced neurotoxicity is suppressed by Ac-DEVD-CHO, a caspase-3 inhibitor, in rat cerebellar granule neurons, suggesting that HNE-induced neuronal death is attributable to activation of the caspase-3-dependent pathway [93]. HNE affects in particular mitochondrial function, signal transduction, transport function, cytokine production and the cytoskeleton [117]. Interestingly, a pre-treatment with high concentrations of NAC completely suppresses the formation of HNE-modified protein, mitochondrial injury and neuronal death [8]. This protective effect is due to an increase of GSH-HNE conjugation by increased GSH levels after treatment with NAC. This highlights the potential of NAC for neurodegenerative disorders.

### IV.2. Current Pharmaceutical Trials 

Several human trials have been launched these last years, especially in FRDA (Table **12**). The most promising drugs are discussed.

#### Idebenone

**Fig. (4):**
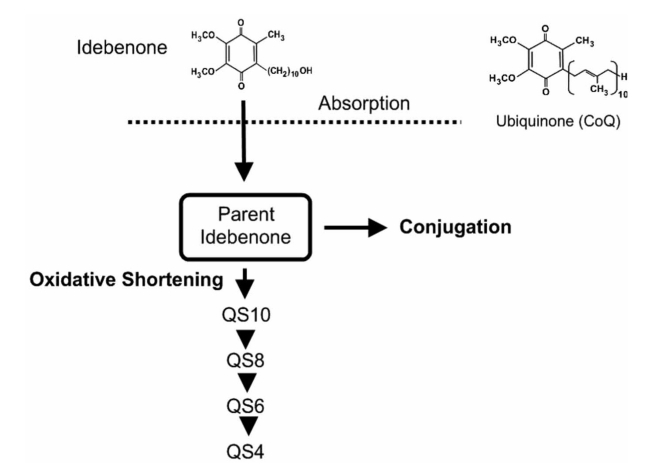
**Schematic rappresentation of the metabolism of Idebenone.** Idebenone is absorbed and can be converted *via* the oxidative shortening or directly being conjugated. The structure of ubiquinone is shown on the upper right corner.

Idebenone is an organic compound of the quinone family and promoted commercially as a synthetic analog of CoQ10. Idebenone is claimed to have properties similar to CoQ10 in terms of antioxidant effects [144]. Idebenone improves deficiencies in electron flow and reduces oxidative stress [120]. Parent idebenone is rapidly metabolized by oxidation and side chain shortening to the inactive metabolites QS10, QS8, QS6 and QS4 (Fig. **4**). Both parent idebenone and the metabolites may be conjugated to sulfates and glucuronides. Phase 1 studies (doses ranging from 150 mg to 1050 mg, either as single oral dose or repeated doses) have shown very low concentrations of the parent drug, with high inter-subject variability [40]. Cmax varies from 1.64 to 23.6 ng/ml according to studies, and tmax ranges from 0.87 to 3.37 hours. Total QS4 is the main metabolic fraction of idebenone in plasma. The pharmacokinetics remain linear for doses ranging from 150 mg to 750 mg daily. The bioavailability is slightly increased when the drug is given after a fat-rich meal. Idebenone is used in Europe in the treatment of vascular and degenerative diseases of the central nervous system [144, 107]. The drug has been used recently in the treatment of FRDA [188]. In a one-year trial on a small number of patients, idebenone reduced the rate of deterioration of cardiac function, without blocking the progression of ataxia [144]. Results of a 3- to 5-years trial suggest that idebenone (1) prevents the progression of cardiomyopathy in both pediatric and adult patients, and (2) stabilizes the neurological dysfunction in pediatric subjects. EPI-A0001 (Edison Pharmaceuticals, San Jose, CA, USA), a bioisostere of CoQ10, is entering in clinical trials [194]. Penwest Pharmaceuticals has just initiated a phase I clinical trial (that will be conducted in healthy volunteers) to evaluate its safety and tolerability at various doses and to collect pharmacokinetic data.

#### Erythropoietin (EPO)

Clinical trials have started, with encouraging results [25]. In one pilot clinical trial, a reduction in oxidative stress markers such as urinary 8-hydroxydeoxyguanosine and serum peroxides has been observed after 5, 000 IU rhu-EPO administration for 8 weeks, three times a week. The frataxin increase observed in this trial is 27%, with high variability between patients (ranging from 15% to 63%). Some non-responders have been identified. However, considering the commercial availability of EPO as drug with assessed safety, these results are promising. Further longer trials are required.

#### Deferiprone (DFP)

**Fig. (5):**
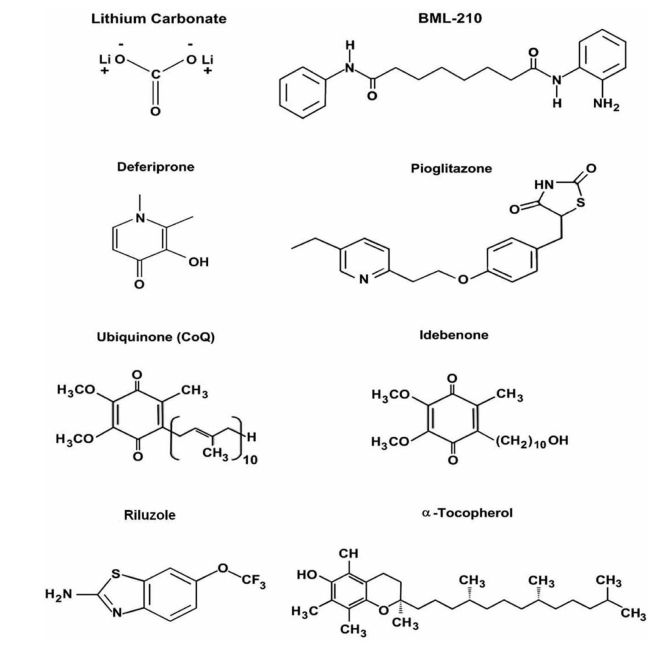
Molecular structures of current drugs assessed for therapy of cerebellar disorders.

For systemic iron overload diseases, like hemochromatosis, the use of iron chelators (desferal or deferiprone DFP, Fig. **5**) has an undeniable relevance [24, 170]. However, the possible role of iron chelators for the treatment of FRDA and other neurodegenerative conditions is still largely controversial [127, 195]. The main point of discussion is the vagueness of the molecular targets of the actual iron chelating drugs. DFP acts as a siderophore to chelate both cytosolic and mitochondrial labile iron. DFP redistributes iron between cellular compartments and to different cell populations, including erythroid precursors, to be used for other metabolic purposes, such as the heme biosynthesis. DFP, as any other clinically relevant chelator, should be used with moderation to avoid overchelation that could affect normal cellular iron metabolism and thereby induce iron deficiency anemia. The importance of exercising moderation in applying chelation relates not only to drug dosage but also to drug exposure time, as dictated by drug pharmacokinetics.

#### Lithium Salts

Lithium carbonate, a compound commonly used to treat manic depression, might also provide symptomatic relief for a group of inherited movement disorders such as SCA1. Treatment with lithium salts reduce motor control difficulties in the mouse model of SCA1. The drug might slow down degenerative changes in the brain. Starting from this important finding, the National Institutes of Health Clinical Center (CC) has started a phase I clinical trial (first patient recruited in March 2009). It should be pointed out that this drug is potentially cerebellotoxic and that a close monitoring is required. Irreversible cerebellar syndromes have been reported.

#### Riluzole

Riluzole preferentially blocks tetrodotoxin (TTX)-sensitive sodium channels which are associated with damaged neurons [197]. This results in a reduction of calcium influx and prevents indirectly the stimulation of glutamate receptors. Together with a direct glutamate receptor blockade, the deleterious effect of glutamate on motor neurons is greatly reduced. However, the action of riluzole on glutamate receptors has been controversial, as no binding of the molecule has been shown on any known receptor [13, 53, 231]. A phase II trial for multiple sclerosis and inherited ataxias is ongoing to assess efficacy of riluzole (S.Andrea Hospital Rome, Italy). 

#### Valproic Acid

Valproic acid (VPA) is primarily used for the treatment of epilepsy, bipolar disorder, and major depression. It is also prescribed to treat headaches and schizophrenia. Valproate may affect neurotransmitter GABA function (as a GABA transaminase inhibitor) in the human brain, making it an alternative to lithium salts in treatment of bipolar disorder. In addition, Valproate may also reverse the transamination process to form more GABA. Hence, VPA acts indirectly as a GABA agonist. However, several other mechanisms of action have been proposed [176]. In particular, it might act as a histone deacetylase 1(HDAC1) inhibitor. A phase I trial has begun in China for SCA3 patients.

#### PPAR-γ Agonists

PPAR-γ agonists, such as rosiglitazone and pioglitazone, are commonly used for the treatment of diabetes mellitus [171, 172] and represent also a promising therapeutic strategy for other diseases including neurodegenerative disorders with an inflammatory component such as Alzheimer’s disease, Parkinson’s disease, multiple sclerosis, and amyotrophic lateral sclerosis (ALS) [78, 79]. Interestingly, this class of molecule has been proposed for mitochondrial diseases, because of their ability to increase the response to oxidative stress, and to act upon mitochondrial biogenesis [1, 37]. A phase II/III trial has started at the Hôpital Robert Debré (France) for FRDA.

## V. THERAPIES UNDER DEVELOPMENT 

New drugs are currently tested in transgenic models of CAs. These new strategies are summarized in Table **13**. In particular, therapeutic strategies which have been successful for Huntington’s disease are being considered for SCAs. This is the case for RNAi therapies which aim to inhibit polyglutamine-induced neurodegeneration. Prevention of protein misfolding and aggregation by overexpressing chaperones, such as heat-shock protein HSP70 or DNAJ1 are being evaluated [87]. HDAC inhibitors can revert silent heterochromatin to an active chromatin conformation, and thus restore the normal function of genes which are silenced in these diseases [47, 80, 174]. Today, the precise site of action of these inhibitors is still unknown. Moreover, it is of great relevance to assess the safety/toxicity of these compounds. Nevertheless, this innovative approach remains one of the most interesting options for the treatment of diseases such as FRDA or FXTAS in which heterochromatin mediating gene silencing occurs. Drugs modulating the cannabinoid receptors and nitric oxide pathways are at a very early stage, and require additional efforts to bring them to the pre-clinical route. Another approach under development is the use of neurotrophic factors promoting survival of Purkinje cells. Two factors play a key-role in this function: glial derived neurotrophic factor (GDNF) and insulin-like growth factor-1 (IGF-1), synthetized by Bergmann glia and Purkinje cells/inferior olive, respectively [152]. GDNF promotes both survival and differentiation of Purkinje neurons, and has shown a protective effect in several models of excitoxicity [4, 210]. Intra-ventricular administration of GDNF protects the shaker mutant rats from loss of Purkinje cells [210]. IGF-1 exerts also neuroprotective effects. In particular, IGF-1 promotes reinnervation and functional recovery after inferior olive lesion [61]. Another potential approach is based on the modulation of microglial activation which follows focal brain damage [225]. Activated microglial cells participate in the process of remote cell death by producing proinflammatory cytokines, nitric oxide, glutamate and free radicals [23]. Minocycline is a tetracycline derivative which reduces microglial activation in axotomized precerebellar nuclei by modulating the inflammatory response consecutive to a cerebellar injury. There is some hope that acting on this mechanism may provide benefits for CAs in the future.

Cerebellum is a target of numerous hormones [116, 217]. It is established that hormonal defects induce cerebellar lesions and that hormones interact with dendritic growth, spinogenesis and synaptogenesis [216]. Surprisingly, very few experimental studies have attempted to take advantages of these effects. The use of hormones for therapy of ataxias probably deserves more attention. 

Transplantation of stem cells is another approach under development, but will not be discussed here for a matter of clarity. Regarding gene therapy and the use of viral vectors, recent advances have contributed to selective and efficient gene transfer to Purkinje cells *in vivo* [83]. A promising application of viral vectors is the rescue of a deficient gene, followed by functional recovery [103]. This strategy might become an appealing approach for therapeutic targeting of disorders affecting Purkinje neurons in the coming decades.

## VI. OVERVIEW OF THE SITES OF ACTION OF THERAPIES OF CEREBELLAR ATAXIAS

**Fig. (6):**
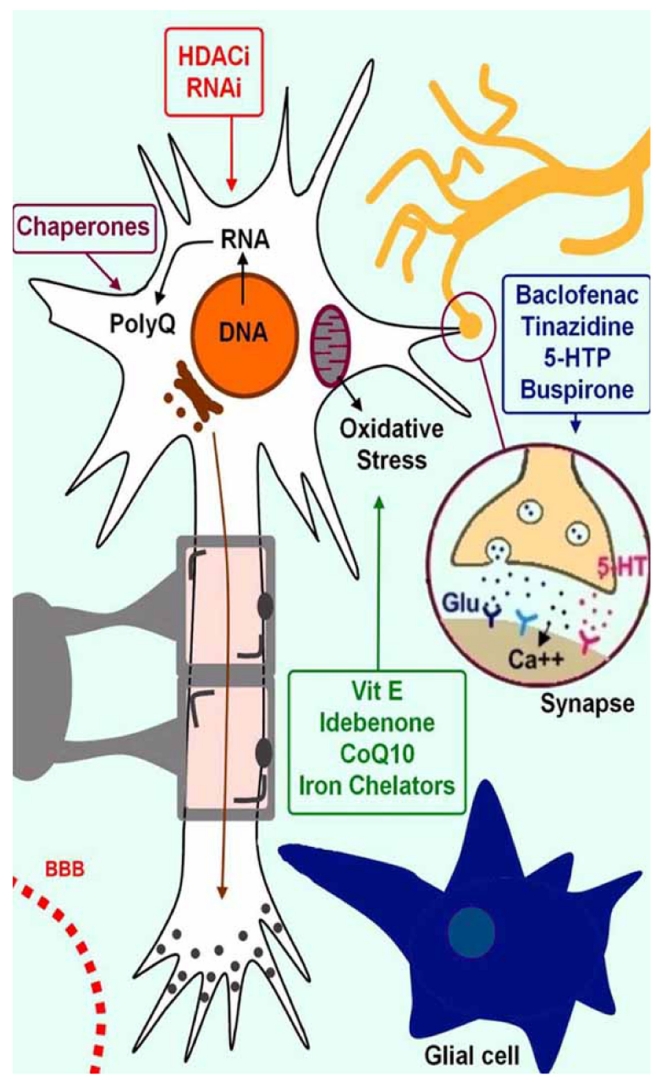
**Illustration of the sites of action of anti-ataxic drugs.** Drugs can be gathered in 4 groups according to the mechanism of action: modulation of synaptic activity, action against oxidative stress, acting on the DNA/RNA level, and targeting the clearance of specific proteins.

The topic of therapeutics of CAs has been considered as a “black box” these last 3 decades. Sets of therapies are being evaluated and it is now possible to draw the lines of therapies in this category of so-called “untreatable neurological disorders” (Fig. **6**):

- drugs acting at synaptic level
- drugs targeting the oxidative stress
- drugs acting on the DNA or RNA
- drugs aiming to decrease the synthesis of abnormal proteins or to increase their clearance.

## VII. CONCLUSION

Our understanding of the pathogenesis of CAs has increased dramatically these last 15 years. Unraveling of genetic defects and developments of animal models are reshaping the neuropharmacology of CAs. For the first time, drugs under development and ongoing clinical trials aim to restore cerebellar function by acting on a deleterious pathway. Several avenues of research are still to be explored. One illustrative example is the therapeutics of neuropsychiatric symptoms recently characterized in cerebellar patients. As a result of the functional heterogeneity of the cerebellar circuitry, it is plausible that drugs under development may selectively improve some cerebellar deficits, whereas other therapies would improve the full clinical deficits. 

There is an obvious medical need to develop anti-ataxic drugs with proved efficacy. The identification of the molecular cascades leading to neuronal death in cerebellar circuitry will greatly help in this endeavor. Such knowledge is required to develop potential therapeutic agents in order to prevent neuronal dysfunction and neurodegeneration.

## Figures and Tables

**Table 1:** Main Clinical Deficits in Cerebellar Patients

Vermal Zone	Paravermal Zone	Lateral Zone

Oculomotor deficits	Dysarthria	Oculomotor deficits
Dysarthria		Dysarthria
Head tilt		Dysmetria
Ataxia of stance/gait		Kinetic tremor
		Hypotonia
		Dysdiadochokinesia
		Decomposition of movements
		Ataxia of stance/gait

**Table 2:** Neuropsychiatric Symptoms in Cerebellar Disorders

Domain	Symptoms

Attention	Distractibility
	Hyperactivity
	Compulsive behaviour
	Perseveration
	Difficulties for shifting attention
	Obsessional behavior

Emotion	Impulsiveness, disinhibition
	Anxiety, agitation
	Pathological laughing and crying
	Anhedonia
	Depression
	Dysphoria

Social skill set	Aggression
	Irritability
	Passivity
	Difficulties with social interactions

Psychosis	Illogical thinking
	Hallucinations
	Lack of empathy

Autism spectrum	Stereotypies
	Avoidant behavior
	Sensory overload

Adapted from Schmahmann and Pandya (2008) [182].

**Table 3:** Differential Diagnosis of Cerebellar Ataxias in Young Adults

Spinocerebellar ataxia (SCA)
Episodic ataxia (EA)
Friedreich ataxia (FRDA)
Wilson disease
Primary tumor
Paraneoplastic ataxia
Infectious/para-infectious
Immune ataxia
Toxics (alcohol)
Endocrine diseases
Leukodystrophies
Mitochondrial disorders

**Table 4:** Main Neurotransmitters and Neuromodulators of Cerebellar Circuitry

Glutamate
Aspartate
GABA
Glycine
Taurine
Amines (Serotonin, Noradrenaline, Acetylcholine, Dopamine, Histamine)
Nitric oxide
Peptides
Endocannabinoids

**Table 5:** Sites of Projections of Aminergic Pathways in the Cerebellum

Amine	Site of Projection in the Cerebellum

Serotonin	Cerebellar cortex: dense plexus in granular and Purkinje cell layer
	Cerebellar nuclei: dense plexus

Noradrenaline	Cerebellar cortex: around glomeruli and around dendrites of Purkinje cells
	Cerebellar nuclei

Acetylcholine	Cerebellar cortex
	Cerebellar nuclei

Dopamine	Cerebellar cortex

Histamine	Cerebellar cortex

**Table 6:** Gene Mutations in Inherited Ataxias

Autosomal Dominant Spinocerebellar Ataxias		
Type	Gene Mutation	Protein
SCA1	CAG (35-83)	Ataxin-1
SCA2	CAG (34-750)	Ataxin-2
SCA3	CAG (56-86)	Ataxin-3(MJD1)
SCA4	-	-
SCA5	Missense mutations	Spectrin
SCA6	CAG (19-33)	Calcium channel, voltage-dependent, P/Q, α-A subinit
SCA7	CAG (41-306)	Ataxin-7
SCA8	CTG (80-300)	Ataxin-8
SCA10	ATTCT (800-4500)	Ataxin-10
SCA11	TTBK2 (1329insA, 1284_1285delAG)	Tau Tubulin kinase-2
SCA12	CAG (66-93)	Protein phosphatase 2, regulatory subunit B, β-isoform
SCA13	KCNC3 (R420H, F448L)	Voltage-gated potassium channels, shaw-related subfamily, member 3
SCA14	Missense mutations	Protein kinase C, g-polypeptide
SCA15	ITPR1 (DEL EX1-48, P1059L)	Type 1 inositol 1, 4, 5-triphosphate receptor
SCA16	-	-
SCA17	CAG/CAA (43-63)	TATA box-binding protein
SCA18	-	-
SCA19	-	-
SCA20	-	-
SCA21	-	-
SCA22	-	-
SCA24	-	-
SCA25	-	-
DRPLA	CAG (48-93)	Atrophin-1
SCA27	FGF 14	Fibroblast growth factors
SCA31	PLEKHG4 (16C-T)	Puratrophin-1
FRDA	9q13	Frataxin
Familial coenzyme Q10(CoQ10) deficiency	CoQ10	CoQ10
Autosomal-recessive spastic ataxia of Charlevoix-Saguenay (ARSACS)	13q12	Sacsin
Mitochondrial recessive ataxic syndrome (MIRAS)		Polymerase-γ
Marinesco-Sjögren syndrome	5q32	SILI
Ataxia with isolated vitamin E deficiency (AVED)	8q13	Alpha-tocopherol transfer protein (α-TTP)
Abetalipoptoteinemia(Bassen-Kornzweig syndrome)	4q22-q24	Microsomal triglyceride transfer protein
Hereditary motor and sensory neuropathy type IV (HMSM IV), Refsum disease	10pter-p11.2	Phytanoyl-CoA hydroxylase
Cerebrotendinous xanthomatosis	2q33-qter	Cytocrome P-450, subfamily VVVIIA, polypeptide 1 (sterol 27-hydroxylase)
Metachromatic leucodystrophy	22q13	Arylsulfatase 1
Niemann-Pick type C	18q11-121	NPC1 protein
GM2-gangliosidosis (Tay-Sachs disease)	15q23-24	Hexosaminidase 1
Chorea-acanthocytosis	9q21	Chorein
Wilsons disease	13q14-21	ATPase Cu transporting beta-polypeptide
Aceruloplasminaemia	3q23-q24	Ceruloplasmin
Ataxia telangiectasia	11q22.3	ATM
Ataxia-telangiectasia-like disorder (ATLD)	11q21	MREIIA
Ataxia with oculomotor apraxia 1 (AOA1/EAOH)	9p13	Aprataxin
Ataxia with oculomotor apraxia 2 (AOA2)	9q34	Senataxin

**Table 7:** Clinical Presentations of Spinocerebellar Ataxias

**Purecerebellar syndrome**	SCA5, SCA6, SCA11, SCA26
**Cerebellar ataxia plus:**	
Cognitive impairment/ behavioural symptoms	SCA1, SCA2, SCA3, SCA10, SCA12, SCA13, SCA14, SCA17, SCA19, SCA21, SCA-FGF14, DRPLA
**Seizures**	SCA10, SCA17, DRPLA
**Eyes/oculomotor deficits**	
Slow saccades	SCA1, SCA2, SCA3, SCA7, SCA28
Down-beat nystagmus	SCA6
Ophthalmoparesia	SCA1, SCA2, SCA3, SCA28, SCA30
Ocular dyskinesia	SCA10
Pigmentary retinopathy	SCA7
**Movement disorders**	
Parkinsonism	SCA1, SCA2, SCA3, SCA12, SCA17, SCA21
Dystonia	SCA3, SCA14, SCA17
Tremor	SCA8, SCA12, SCA16, SCA19, SCA20
Dyskinesias	SCA-FGF14
Myoclonus	SCA2, SCA14, SCA19, DRPLA
Chorea	SCA1, SCA17, DRPLA
Myokymia	SCA5
**Pyramidal signs**	SCA1, SCA2, SCA3, SCA4, SCA7, SCA8, SCA10, SCA11, SCA12, SCA13, SCA14, SCA15, SCA28, SCA30
**Peripheral neuropathy**	SCA1, SCA2, SCA3, SCA4, SCA6, SCA8, SCA-FGF14, SCA12, SCA18, SCA22, SCA25

**Table 8:** Genetics of Episodic Ataxias (EAs)

Type of Ataxia	Gene Mutation and Function
Type 1(EA-1)	KCNA1-deficiency in voltage-gated potassium channel function Autosomal dominant
Type 2 (EA2)	CACNA1A-subunit of P/Q type calcium channel; different mutations in same gene lead to SCA6 and familial hemiplegic migraine CACNB4-dihydropyridine-sensitive L-type calcium channel Autosomal dominant
Type 3(EA-3)	Linked to 1q42
Type 4 (EA-4)	Unknown
Type 6 (EA-6)	Unknown
Type 7 (EA-7)	19q13

Adapted from Brust (2006) [31].

**Table 9:** Main X-Linked Ataxias

Fragile X tremor ataxia syndrome (FXTAS)
Arts Syndrome
X-linked adrenoleukodystrophy (X-ALD)
Congenital ataxias
Rett syndrome
Ataxia-Dementia (SCAX4)

**Table 10:** Acquired Ataxias

Stroke (infarction, haemorrhage)
Toxic-induced (ethanol, heavy metals, solvents, drugs)
Immune-mediated
Infectious/parainfectious diseases (abscess, cerebellitis)
Traumatic
Context of neoplastic disorder (Primary cancer, metastases, chemotherapy, stroke, paraneoplastic)
Endocrine (hypothyroidism)
Structural disease (Chiari malformations, agenesis, hypoplasias, dysplasias).

**Table 11:** Main Drugs which May Trigger Cerebellar Ataxia

Lithium salts
Phenytoin
Valproate
Amiodarone
Metronidazole
Procainamide
Calcineurin inhibitors
Mefloquine
Isoniazid

**Table 12:** Results of Clinical Trials in Spinocerebellar Ataxias (SCAs) Caused by CAG Repeats

Disease Ref	Treatment	Design of the Trial	Results
SCA3 [38]	Sulfamethoxazole Trimethoprim	Double-blind, placebo controlled crossover	Improved gait and coordination
SCA3 [187]	Sulfamethoxazole Trimethoprim	Double-blind, placebo controlled crossover	No effect
SCA3 [178]	Tetrahydrobiopterin	Double-blind crossover	Improvement on timed tests
SCA6 [233]	Acetazolamide	Open label	Improvement on Ataxia Rating Scale
SCA3 [204]	Tandospirone	Open label	Improvement on Ataxia Rating Scale

From Underwood and Rubinsztein (2008) [219].

**Table 13:** Therapeutical Trials in Cerebellar Ataxias

FRIEDREICH’S ATAXIA
Coenzyme Q10 (CoQ10) with E-pilot study
Idebenone - Phase III
Mitoquinone (MitoQ) - Phase II
Erythropoetin (EPO) - pilot, PhaseI/II
Chelation therapy (Deferiprone) - Phase I/II
EPI-A0001 (RAID program)-pre-drug
HDAC inhibitors - pre-drug
PPARgamma agonists - Phase II/III
Varenicline - Phase II
SPORADIC ATAXIAS
Gluten-free dietpilot study
Immunomodulation (IV immunoglobulins, plasmapheresis) -pilot study

**Table 14:** Drugs Targetting A Deleterious Pathway

Acting against oxidative Stress and Free Radicals (Mitochondria)
Vitamin E
Coenzyme Q10 (CoQ10)
Creatine
Alpha lipoic acid
Tauroursodeoxycholic acid (TUDCA)
Ethyl-EPA (LAX-101)
Apoptosis/Excitotoxicity
Anti-glutamate (*remacemide, riluzole, memantine*)
Anti-caspase-1 (*minocycline, ethyl-EPA*)
“Declumping”-reducing fragment load
Anti-caspase 3 (*minocycline* and others)
Anti-caspase 6
Anti-transglutaminase (cystamine)
“Declumping”-reducing aggregation
Inhibition of GSK-3beta (*lithium chloride, trehalose*)
Transcriptional re-regulation-upregulating expression of genes inhibited by mHtg.
Valproic acid
SAHA/suberoylanilide hydroxamic acid,
Phenylbutyrate
Transcriptional hijacking-small inhibitory RNAs to block the production of mHtg.
